# Rheumatic Manifestations in Patients Treated with Immune Checkpoint Inhibitors

**DOI:** 10.3390/ijms21093389

**Published:** 2020-05-11

**Authors:** Konstantinos Melissaropoulos, Kalliopi Klavdianou, Alexandra Filippopoulou, Fotini Kalofonou, Haralabos Kalofonos, Dimitrios Daoussis

**Affiliations:** 1 Department of Rheumatology, Agios Andreas General Hospital, 26335 Patras, Greece; knmelis@gmail.com; 2 Department of Rheumatology, “Asklepieion” General Hospital, 16673 Athens, Greece; polyklavdianou@gmail.com; 3 Department of Rheumatology, Patras University Hospital, University of Patras Medical School, 26500 Patras, Greece; alexfilippop@yahoo.com; 4 SHO-Clinical Fellow in Medical Oncology, Imperial Healthcare NHS Trust, London W21NY, UK; kalfotini@gmail.com; 5 Department of Oncology, Patras University Hospital, University of Patras Medical School, 26500 Patras, Greece; kalofonos@upatras.gr

**Keywords:** immune checkpoint inhibitors, cancer immunotherapy, rheumatic, musculoskeletal, arthritis, myositis, polymyalgia rheumatica, systemic lupus erythematosus, sicca, scleroderma

## Abstract

Immune checkpoint inhibitors (ICIs) are monoclonal antibodies that activate the immune system, aiming at enhancing antitumor immunity. Their clinical efficacy is well-documented, but the side effects associated with their use are still under investigation. These drugs cause several immune-related adverse events (ir-AEs), some of which stand within the field of rheumatology. Herein, we present a literature review performed in an effort to evaluate all publicly available clinical data regarding rheumatic manifestations associated with ICIs. The most common musculoskeletal ir-AEs are inflammatory arthritis, polymyalgia rheumatica and myositis. Non-musculoskeletal rheumatic manifestations are less frequent, with the most prominent being sicca, vasculitides and sarcoidosis. Cases of systemic lupus erythematosus or scleroderma are extremely rare. The majority of musculoskeletal ir-AEs are of mild/moderate severity and can be managed with steroids with no need for ICI discontinuation. In severe cases, more intense immunosuppressive therapy and permanent ICI discontinuation may be employed. Oncologists should periodically screen patients receiving ICIs for new-onset inflammatory musculoskeletal complaints and seek a rheumatology consultation in cases of persisting symptoms.

## 1. Introduction

The notion of immune system manipulation to achieve antitumor effect entails decades of basic research effort, but it has only recently achieved broad clinical implementation in the field of oncology. Better understanding of tumor genetics and immune surveillance mechanisms is necessary to fight cancer in a more efficient and effective way [1]. While our immune system recognizes cancer cells, it is restrained by various “checkpoints”; molecules such as cytotoxic T lymphocyte antigen 4 (CTLA4), programmed death 1 (PD-1) and its ligand PD-L1 act as brakes restricting T cell effector functions. This process is important for homeostasis and autoimmunity prevention in healthy organisms, but on the other hand it dampens critical T cell cytotoxic functions against tumor cells in cancer patients. Immune checkpoint inhibitors (ICIs) are monoclonal antibodies which target checkpoint molecules and have significant clinical efficacy, rendering immune checkpoint blockade an emerging therapeutic approach in cancer [2]. There is a major expansion in the number of clinical trials involving multiple immunotherapy agents in a variety of cancer types, with lung cancer, melanoma, breast cancer, lymphoma and head and neck cancer being the most studied ones [3].

The widespread implementation of ICIs over the last decade has provided important data on their toxicity profile [4]. The attenuation of T cell inhibitory mechanisms by ICIs leads to hyperactivation of the immune system; as probably expected, this associates with a variety of adverse events characterized by inflammation. Target sites of these adverse events, usually termed as immune-related adverse events (ir-AEs) can include every tissue in the human body, including the gastrointestinal tract, endocrine glands, liver and skin, while cardiovascular, pulmonary and rheumatic ir-AEs are also reported [5].

In this review, rheumatic manifestations in the context of ICI therapy will be discussed. Musculoskeletal and non-musculoskeletal clinical manifestations will be separately analyzed, along with current data concerning imaging and treatment.

## 2. Methods

We performed an electronic search (PubMed) covering up until March 2020 using the keywords “immune checkpoint inhibitors” or “cancer immunotherapy” combined with “arthritis”, “myositis”, “polymyalgia rheumatica”, “musculoskeletal”, “rheumatic”, “sicca”, “vasculitis”, “sarcoidosis”, “systemic lupus erythematosus” and “systemic sclerosis” in all possible combinations. Only papers published as full articles in the English language were included, and no time limit was set. We supplemented the computerized search with a manual one of the reference lists of the retrieved articles. The abstracts of all retrieved articles were assessed in order to identify reports related to rheumatic manifestations in patients treated with ICIs.

## 3. Results

### 3.1. Musculoskeletal Immune-Related Adverse Events

Three main clinical phenotypes induced by cancer immunotherapy have been described in the oncology and rheumatology literature: inflammatory arthritis, myositis and polymyalgia-like syndrome [6,7]. The pathophysiology of these ICI-induced rheumatic manifestations needs further clarification, since these syndromes appear to have differences from the respective idiopathic rheumatic diseases. Crucial questions arise, including how these rheumatic manifestations should be treated, whether ICI therapy should be discontinued and if patients should be re-challenged in case of discontinuation [7].

#### 3.1.1. Inflammatory Arthritis

Symptoms from joints appear to be the commonest musculoskeletal complaint among patients receiving ICI therapy. In a systematic review of the literature from 2017 [8], joint pain was reported to occur in a wide range of 1%–43% of participants exposed to ICIs in clinical trials. Mild arthralgia appears to be a relatively common symptom among patients treated with ICIs; it usually responds well to analgesics and does not seem to have any clinical significance. However, a minority of patients develop more pronounced pain with inflammatory features, such as morning stiffness as well as joint swelling suggestive of arthritis. Arthritis prevalence does not seem to exceed 7% [9]. Terminology and heterogeneity issues exist, making estimation of true prevalence difficult. Data from three prospective studies addressing musculoskeletal complications in ICI-treated patients [10,11,12] report inflammatory arthritis in approximately 2%–3% of the cases. In these studies, clinical presentation included not only a rheumatoid arthritis (RA)-like pattern with symmetrical polyarthritis involving wrists and hands, but also cases of oligoarthritis of the lower limbs and cases of psoriatic arthritis. Median time to symptom onset following ICI initiation did not exceed the period of three months. Review of the literature, however, highlights the clinical variability of ICI-exposed patients developing arthritis [9,13]. In a recent review of case reports and case series [14], 90 cases of de novo ICI-related arthritis were identified in a period of 18 months. A few patients developed well-differentiated clinical syndromes such as RA, fulfilling classification criteria [15], and psoriatic arthritis. Most cases, however, developed undifferentiated arthritis without a specific clinical pattern. Monoarthritis, usually involving the shoulder, along with oligoarthritis and polyarthritis were reported. These cases occurred with anti-PD1/PDL1 or combination ICI therapy. Median time to symptom onset, especially in cases of RA, PsA, RA-like and polyarthritis presentation, was usually a few months following treatment initiation. Besides the criteria-fulfilling RA patients, who tested all positive for rheumatoid factor (RF) and/or anti-cyclic citrullinated peptide (CCP) antibodies, the other cases were mostly seronegative. Case reports of remitting seronegative symmetrical polyarthritis with pitting edema (RS3PE) [16,17,18,19] and patients with a reactive arthritis (ReA)-like syndrome with concurrent urethritis and conjunctivitis [20] extend the spectrum of clinical phenotypes. A study by Cappelli et al. [21] provides interesting data linking combination ICI therapy with a more severe disease course and distinct clinical features compared to anti-PD1/PDL1 monotherapy. In a more recent prospective study, combination ICI treatment schemes, longer duration of ICI therapy and the history of ≥2 ir-AEs also predicted a more persistent course of arthritis [22]. In this study, almost half of the patients exhibited active inflammatory arthritis six months after immunotherapy cessation. The pleiomorphy of ICI-related joint symptoms should be taken into consideration at the time of clinical differential diagnosis of arthritis in these patients, which could also include paraneoplastic syndromes, cancer cell invasion of the joint and degenerative joint disease.

Imaging is a useful tool for the more detailed depiction of arthritis in the context of ICI treatment, in addition to the exploration of alternative diagnoses. A retrospective sonographic study [23] involving nine patients showed evidence of synovitis in 12/18 joint regions assessed, active Doppler signal in 9/18 and tendon involvement in the form of tenosynovitis or enthesophytes in 13/18. Erosions were detected in three joints. Structural findings, such as erosions and enthesophytes, were found in patients with symptoms of only a few months, implying that these could be early onset changes. Synovial fluid analysis was performed, in some cases revealing an inflammatory aspirate. One patient in the study was found to have metastatic joint disease, indicating the importance of ruling out metastasis in all cases of patients with cancer developing specific musculoskeletal symptoms. Magnetic resonance imaging (MRI), has also been used to evaluate ICI-induced arthritis. In our department [10], we performed the first prospective study to systemically report MRI data concerning ICI-induced musculoskeletal manifestations. Three distinct clinical patterns were evident: prominent joint involvement, prominent periarticular involvement and myo-fasciitis. Interestingly, imaging data from most patients in all groups revealed pathologic signal in surrounding muscles and fascia, indicating that these structures were more frequently involved than the synovium. Histological findings of a single patient from the myo-fasciitis group showed a chronic inflammatory infiltrate consistent with the MRI findings. Our data deriving from imaging and histology place myo-fasciitis as a central pathophysiologic feature of ICI-induced musculoskeletal manifestations and could pinpoint a subgroup of patients with arthritis-like presentation, mostly involving, however, tendons and myo-fascia. In a subsequent study reporting MRI data in ICI-induced inflammatory arthritis [24], eight patients were retrospectively evaluated. Tenosynovitis and synovitis of hands and wrists was a common finding, while a few patients were also found to have osseous erosions indicating a worse prognosis.

ICI-induced joint symptoms are usually mild or moderate in severity and ICI treatment can be continued in most cases. The decision for ICI discontinuation should also take into account the co-existence and severity of other non-rheumatic ir-AEs, present almost in half of the patients in the study by Pundole et al. [14]. Patients with mild arthralgia require only analgesic treatment; specific investigations and a rheumatology consultation is not necessary in these cases. However, patients with new-onset intense joint pain associated with morning stiffness or non-traumatic joint swelling should be referred to a rheumatologist. Most patients with ICI-induced arthritis can be managed with low/moderate dose of steroids without need for ICI discontinuation. In our department, we administer 12 mg of methylprednisolone with gradual tapering according to clinical response within a period of approximately 3–6 months; the use of this particular scheme exhibits a favorable response for most patients. In case of arthritis relapse during steroid tapering, a synthetic disease-modifying antirheumatic drug (DMARD) could be added either in the form of methotrexate (MTX) or hydroxychloroquine (HCQ). Besides synthetic DMARDs, biologic therapy targeting TNFα has been infrequently reported in more severe cases [21]. Functional disability, combination ICI therapy, RF and/or anti-CCP positivity, distinct clinical syndromes (such as RA, PSA or ReA) and imaging findings of erosions are among the features that could warrant a more aggressive therapeutic approach.

#### 3.1.2. Polymyalgia Rheumatica

Classic polymyalgia rheumatica (PMR) is characterized by proximal muscle pain, usually without definite synovitis, typically involving the areas of upper arms, neck, shoulders, hips and thighs. Morning stiffness and fatigue are common complaints. Age over 50 years old and increase in inflammatory markers, in the absence of typical RA autoantibodies, are considered essential features for diagnosis [25]. A similar syndrome is encountered in patients receiving ICIs, as indicated in various case series [11,12,26,27,28,29,30,31]. In the largest prospective study until now [11], 11/524 patients who started ICI therapy developed a new-onset PMR-like syndrome, providing an estimated prevalence of 2.1%. A prospective study of 210 patients [12] and a large retrospective study in Mayo clinic [26] provide data that indicate a somewhat lower prevalence of the syndrome, below 1%. Calabrese et al. collected 49 reported cases of PMR-like syndrome in patients on ICI therapy, analyzing data from three major rheumatology centers from USA and Europe along with a systematic review of the literature [32]. It is noteworthy that 25% of the cases did not fulfill the preliminary 2012 European League Against Rheumatism/American College of Rheumatology (EULAR/ACR) criteria for PMR. Atypical features were observed, such as the involvement of other joints (mostly knees and hands), the absence of high inflammatory markers and the presence of aggressive cases that are nonresponsive to usual treatment.

Most cases of this PMR-like syndrome appear to occur early, within months of ICI treatment initiation, with both anti-CTLA4 and anti-PD1/PDL1 inhibitors [32]. In the setting of a PMR-like syndrome, it is important to seek symptoms suggesting temporal arteritis, such as headache and visual impairment. This is a standard practice in rheumatology because PMR and temporal arteritis belong to the same spectrum of disorders and frequently co-exist. Albeit rare, cases of PMR and concomitant temporal arteritis following ICI treatment have been published [33]. As far as imaging is concerned, ultrasound analysis [11], MRI [30] and FDG-PET/CT [29] assist diagnosis in certain patients by providing images of subdeltoid bursitis, rotator cuff bursitis/tendinitis and bilateral hip articular/periarticular uptake, respectively.

Idiopathic PMR responds well to doses of prednisolone below 20 mg/day; a good and fast response to steroids strengthens the diagnosis in everyday clinical practice. ICI-induced PMR-like syndrome seems to somehow defy this general rule in about 40% of the cases [32]. Patients requiring more aggressive therapy with higher doses of steroids mostly account for this percentage. We should note, however, that some cases exhibit mild symptoms and respond to non steroidal anti-inflammatory drurs (NSAIDs) lone [11,32]. Three cases have been reported also requiring conventional synthetic DMARDs as steroid sparing agents, while one was treated with infliximab with minimal improvement [30] and another two responded well to tocilizumab [32]. In conclusion, most cases of ICI-induced PMR-like syndrome respond well to moderate doses of steroids, but the clinician should be aware that atypical cases with normal acute phase reactants exist and more aggressive therapy is sometimes required.

#### 3.1.3. Myositis

Idiopathic inflammatory myopathies (IIM) are a heterogeneous group of disorders characterized by clinical signs of muscle weakness and findings indicative of muscle inflammation in neurophysiological evaluation and histopathologic studies. Classic dermatomyositis and certain autoantibodies exhibit a strong association with cancer [34], and in these cases the term paraneoplastic myositis is often used. On the other hand, a de novo condition with features of myopathy/myositis has been described in recent years following ICI therapy in cancer patients [6,9,13]. The differentiation between paraneoplastic and ICI-induced myositis can be sometimes difficult [35]. Prospective studies [10,11,12] report only few cases of ICI-induced myositis, indicating a low prevalence of this condition. The occurrence of ICI-induced myositis, however, seems to rise, probably along with the increase in the number of patients treated with ICIs worldwide. In addition, ICI-induced myositis has atypical features compared to idiopathic forms of the disease and carries a high mortality risk, suggesting that this is the most serious musculoskeletal ir-AE. Analysis of the World Health Organization (WHO) pharmacovigilance database VigiBase [36,37] has identified 345 ICI-exposed myositis cases, with over 150 cases being reported between March 2018 and February 2019. In a recent study by Nguyễn et al. [37] most patients were reported to be male and aged >63 years old; moreover, myositis appeared soon following ICI initiation at a median time of about four weeks and was associated with an increased fatality rate of 22.3%, with almost 95% of the cases requiring hospitalization. All available ICIs were linked to myositis, and in most cases ICI treatment was discontinued. Of note, there was a strong association between ICI-induced myositis and the co-occurrence of myocarditis and myasthenia in 11.3% and 11.9% of the cases, respectively, resulting in increased mortality. Case reports of simultaneous myositis, myasthenia and myocarditis also exist in the literature [38].

Better characterization of myositis-like syndromes is available through case series of patients [39,40,41,42]. Clinical data derived from these studies confirm the early onset of the syndrome following only a few infusions of ICI therapy, as well as the frequent concomitant presence of myasthenia-like features and cardiac involvement. Interestingly, a significant proportion of patients presented with myalgia and concurrent proximal and axial weakness. Of note, myalgia is not a feature of polymyositis, the prototype inflammatory myositis encountered in rheumatology; patients with polymyositis report subacute onset of proximal muscle weakness but almost never report muscle pain. Distal weakness and bulbar and oculomotor symptoms were often reported, the latter contrary to our knowledge that facial and extraocular muscles are almost never affected in patients with IIM [34]. Typical manifestations of IIM, such as skin lesions and interstitial lung disease, were largely absent in these cases. Electrophysiological studies revealed myopathic pattern in all cases. The majority of patients had an increase in creatine phosphokinase (CPK) and other muscle enzymes, but we should underscore the fact that several reports of ICI-induced myopathy with normal muscle enzymes exist [10,41]. Antibodies associated with myositis and myasthenia gravis were negative in most cases. Of note, an ICI-induced myasthenia gravis case series [43] also gives an interesting perspective, since these patients exhibited a more severe disease course, lower frequency of autoantibody (such as the anti-acetylcholine receptor antibodies) positivity and greater proportion of concurrent myositis features compared to idiopathic myasthenia gravis patients. Histopathologic data [39,41,42] reveal a specific pattern: myofiber necrosis, macrophage and T cell infiltration with relative absence of B cells. These findings are also present in the myocardium [42,44,45]. It seems that cases of ICI-induced myositis may have distinct pathogenesis, yet to be fully understood. This pathogenetic mechanism also carries myasthenic and myocarditis-related features, possibly targeting both muscle fibers and neuromuscular junction in a T-cell-driven way and differentiated from classic IIM and idiopathic myasthenia gravis. Increased awareness and appropriate laboratory tests, such as CPK and serum troponin measurement, are especially needed in the first months following introduction of ICI treatment.

MRI is a useful imaging tool, especially in aiding cardiac evaluation and identifying muscle sites affected for a forthcoming muscle biopsy [39]. It is interesting that MRI data from two studies [10,12] report findings of increased signal intensity in both muscle and fascia in patients with myalgia of the lower limbs, thus characterizing a distinct, possibly milder, clinical phenotype [46].

ICI treatment is almost always permanently discontinued for many patients responding, at least partially, to high-dose steroids. It is not clear whether ICI treatment can be re-administered, although such an effort has been attempted in selected cases [47]. In summary, milder myopathy clinical phenotypes with myalgia and low or absent CPK elevation respond well to moderate doses of steroids. However, clinical syndromes with severe functional impairment, major increase in CPK and/or myasthenia features and cardiac involvement warrant definite ICI discontinuation and more aggressive treatment, including high-dose steroids, plasma exchange, intravenous immunoglobulin (IVIG) and immunosuppressants [48].

#### 3.1.4. Association of Immune Checkpoint Inhibitor (ICI)-Induced Musculoskeletal Manifestations and Oncological Response

The occurrence of ir-AEs may suggest that the immune system of the patient is overactivated; based on this, one may hypothesize that an overactive immune system may be more capable in fighting cancer. Indeed, accumulating clinical evidence indicates a potential link between ir-AEs and tumor response [5,49]. Study of this issue requires well-powered prospective studies. Regarding musculoskeletal ir-AEs, prospective trials show that their occurrence correlates significantly with a better oncological response compared to patients who do not exhibit these manifestations [10,11]. This is of potential clinical significance and suggests that collaboration between rheumatologists and oncologists is important; patients with musculoskeletal ir-AEs should be diagnosed and treated early and effectively so that treatment with ICIs can be continued.

### 3.2. Non-Musculoskeletal Rheumatic Manifestations Induced by ICIs

#### 3.2.1. Vasculitis

Large and medium vessel vasculitides have been linked to dampened immune checkpoint function [50,51]. Research in giant cell arteritis (GCA) has revealed dysfunction of immune checkpoints. Dendritic cells generated ex vivo from patients with GCA have been reported to be PDL-1-deficient; on the other hand, vessel-infiltrating T cells express PD-1 [52]. These findings strongly link vasculitis to downregulation of PD-1/PD-L1 pathway, occurring either spontaneously or by ICIs. Additionally, expression of the negative immune checkpoint, V-domain immunoglobulin-containing suppressor of T cell activation (VISTA) in peripheral blood immune cells has been found to be decreased in GCA patients [53]. The decreased expression of the immune checkpoint VISTA may lead to Th1 and Th17 overexpression which may have pathogenetic implications. The above data indicate that vascular inflammation is tightly linked to dysfunctional immune checkpoints. Therefore, it is not surprising that inhibition of immune checkpoints in the context of cancer immunotherapy may lead to vasculitis.

ICI-induced cases of vasculitis have been reported in the literature, mainly in the form of case reports or case series. A systematic review has identified several cases of ICI-induced vasculitis [54]. The term “autoimmune vasculitis” has been used in the phase II study of ipilimumab [55]. Various forms of vasculitis affecting large vessels have been reported, and the corresponding diagnoses include GCA [33,56,57] or periaortitis [58]. Additionally, medium or small vessels can be affected, with cases of granulomatosis with polyangiitis (GPA) [59,60], eosinophilic granulomatosis with polyangiitis (EGPA) [61] and cryoglobulinemic vasculitis [62] having been reported. Cutaneous granulomatous [63,64] and leukocytoclastic vasculitides [65] have also been described. ICI-induced vasculitis can present as isolated vasculitis affecting the central nervous system (PACNs) [66,67,68], the peripheral nervous system [69,70], the retina [71,72,73], the testicles [74] and the uterus [75]. The most frequent malignancy reported with ICI-induced vasculitis is melanoma. The majority of patients were males in the fifth or sixth decade and diagnosis was mostly histologically proven; however, in a few cases, diagnosis was made only by imaging methods. Patients with ICI-induced vasculitis usually do not have autoantibodies, and the main serological finding is elevation of inflammatory markers. ICI discontinuation and immunosuppression with high doses of steroids led to partial or complete resolution of clinical manifestations in the majority of patients. Currently, it is still not known whether ICI-induced vasculitis is associated with a better oncological response, due to the few cases reported.

There are case reports describing acral vasculitis with digital ischemia due to either an ir-AE or a paraneoplastic syndrome [62,76,77,78,79]. Distinguishing ICI-induced vasculitis from paraneoplastic vasculitis can be very challenging, since the latter is sometimes concurrent with malignancies [80]. However, the resolution of vasculitis following ICI discontinuation and steroid treatment could be indicative of ICI-induced vasculitis. In conclusion, cases of ICI-induced vasculitis are well-described but appear to be relatively uncommon. Taking into account that systemic vasculitides may associate with severe clinical manifestations and target organ damage, ICIs should be discontinued and therapy with high-dose steroids should be implemented.

#### 3.2.2. Sarcoidosis and Sarcoid-Like Reactions

Several cases of ICI-induced sarcoidosis or sarcoid-like reactions have been described in the literature, mostly following treatment with anti-CTLA4 or anti-PD1, rather than anti-PDL1 or combination regimens [81]. The pathogenetic mechanism of ICI-induced sarcoidosis is not well understood, but it is of note that decreased expression of CTLA4 in Tregs and Th17 cells has been reported in patients with sarcoidosis [82]. This may lead to defective Treg suppressive function and, on the other hand, to increased activation of Th17 cells; both these events may have pathogenetic implications [83]. These data may at least partially explain the occurrence of sarcoidosis/sarcoid-like reactions following CTLA4 blockade.

New-onset sarcoidosis has been mainly reported in association with melanoma [84], with a prevalence of 0.2% according to the nationwide multicenter registry in France, the Registry of Severe Adverse Reactions to Immunomodulatory Antibodies in Oncology (REISAMIC) [28]. ICI-induced sarcoidosis can present as cutaneous sarcoidosis [85] or systemic with lymphadenopathy, lung [86] or neurological and ocular involvement [87,88,89,90]. There are no specific serum findings for ICI-induced sarcoidosis. Serum angiotensin converting enzyme (SACE) serum levels can be elevated [91] or normal [92,93]. In most cases, sarcoidosis has been managed with temporary ICI discontinuation and steroid treatment [94].

The most common underlying malignancy associated with sarcoid-like reactions is melanoma, with a slight female predominance [81]. The onset of sarcoid-like reactions varies, usually occurring between three weeks and two years following ICI initiation [95], and the most commonly affected organs are the lungs and skin. Interestingly, 10 cases have been reported where sarcoid-like reactions on permanent tattoos were induced by ICI therapy [96]. Sarcoid-like granulomatosis and lymphadenopathy of the thorax has been reported in 5%–7% of ipilimumab-treated melanoma patients [97,98]. Regarding imaging, patients may present with not only mediastinal or hilar lymphadenopathy in CT, but also parenchymal lung CT changes, such as ground glass opacities [99]. This is of major clinical significance since sarcoid-like reactions can be misdiagnosed as disease progression. Lymph node biopsies performed in these cases to exclude cancer recurrence or progression mostly demonstrated non-necrotizing granulomatous inflammation [100]. Functional lung tests performed in some cases showed either a moderate restrictive pattern and reduced carbon monoxide diffusing capacity [93] or were normal [101,102]. Patients developing ICI-induced sarcoid-like reactions were usually asymptomatic and the majority did not mandate permanent ICI discontinuation or systemic treatment [81,94,103], although ICI discontinuation and/or additional steroid treatment became necessary for resolution of granulomatous reactions in some cases. For cutaneous granulomatosis, topical steroids have been proven effective. Sarcoid-like reactions have not been clearly connected with better cancer prognosis [81]. However, a review by Tetzlaff et al. has identified better disease prognosis in 71% of reported melanoma patients who developed granulomatous/sarcoid-like lesions associated with ICIs over a median follow-up of 11.5 months since therapy initiation [104].

### 3.3. Sicca and Sjogren’s Syndrome

Sjogen’s syndrome (SS) is a rheumatic disease clinically characterized by xerophthalmia and xerostomia; from a pathogenetic point of view, it has been described as an autoimmune epithelitis [105,106]. Imbalances in immune checkpoints can induce the activation and proliferation of autoreactive cells that may lead to SS [107]. Polymorphisms of CTLA4 have been correlated with disease susceptibility and autoantibody production [108,109]. Moreover, treatment with a vector encoding CTLA4-IgG fusion protein has been shown to reduce cell infiltration and improve salivary gland function in a mouse model of SS [110]. CTLA4 deletion in murine Tregs resulted in sialadenitis [111], and PDL-1 has been reported to prevent SS development in non-diabetic obese mice [112]. These data indicate a strong link between dysfunction of immune checkpoints and development of sicca. Therefore, cases of ICI-induced sicca were not unexpected. Sicca has been reported as an ir-AE in clinical trials, with an incidence rate ranging from 1.2% to 24.2% [8,113]. Le Burel et al. reported 0.3% prevalence of SS in REISAMIC registry of grade ≥2 ir-AEs [28], yet the precise incidence rates are not known. Several case series of patients with ICI-induced sicca [20,30] have been reported. A recent review identified 17 cases of sicca symptoms following ICI treatment [9]. These patients had a median age of 63 years, with a slight male predominance (53%); most had melanoma (71%) and had received anti-PD(L)1 therapy (88%). Median time to ir-AE following treatment initiation was 3.8 months. Two recent studies have identified 35 more patients developing sicca syndrome associated with ICI therapy [114,115]. Dry mouth symptoms appeared at a median time of 70 days following ICI treatment initiation. The majority of patients had negative antinuclear antibodies (ANA), extractable nuclear antigens (ENA) and RF. Most patients underwent salivary gland biopsies which demonstrated mild-to-severe sialadenitis distinct from SS with diffuse T cell lymphocytic infiltration and acinar injury. Tissue damage was mediated by CD4+ and CD8+ T cell infiltration, but no correlation between clinical symptoms and histological findings was reported. In the majority of cases, ICIs were discontinued either temporarily or permanently, and steroids were administered when the ir-AEs were characterized as grade 3; in some cases, ICI-induced xerostomia can be severe and may lead to feeding difficulties or even to total parenteral nutrition. The ImmunoCancer International Registry reported 26 patients with sicca following anti-PD1/anti-PDL-1 treatment [116]. There was a male predominance in this report, and from a clinical point of view, mouth dryness was more frequent than ocular dryness. Histopathological findings consisted mostly of mild chronic sialadenitis, whereas positive autoantibodies were rare. Therapeutic management included local sicca treatment and steroids in 40% of the cases. Notably, only 62% of the patients with sicca symptoms fulfilled SS classification criteria. The above data indicate that ICI-induced sicca may be a clinical entity distinct from idiopathic SS. Patients with ICI-induced sicca are more often male, in sharp contrast to SS which is virtually a female disease. Dry mouth is the predominant symptom, and in many cases ocular and oral dryness do not coexist; patients with ICI-induced sicca have a low frequency of abnormal ocular tests, and less than half of salivary gland biopsies are typical for SS. Treatment mainly consists of saliva and tear substitutes. In cases of severe xerostomia that causes feeding problems, the use of steroids may be justified.

### 3.4. Systemic Connective Tissue Diseases

#### 3.4.1. Systemic Lupus Erythematosus (SLE)

Dysregulation of immune checkpoints have been involved in SLE pathogenesis. Data suggest that both PD1 and CTLA4 gene polymorphisms in humans and deficiencies in animal models can lead to lupus manifestations [117,118,119]. Despite the fact that SLE is the prototype systemic autoimmune disease, ICI-induced lupus is a very uncommon ir-AE [120]. According to the FDA Adverse Event Reporting System, SLE was reported in only 18 cases until June 2018, plus 7 cases of cutaneous lupus, 2 cases of lupus-like syndrome and 1 case each for lupus nephritis and central nervous system lupus [121]. These cases developed at a median time of 196 days following anti-PD(L)1 treatment. Of note, demographics of these patients had striking differences compared to patients with idiopathic SLE, which is a disease that mostly affects young women. The mean age was 61 years and the female:male ratio was 1.6:1 in contrast to 9:1 in idiopathic SLE. Five more cases of ICI-induced lupus have been reported in the REISAMIC registry, with four out of five patients developing cutaneous lupus, and ANA being positive in only two patients. Most patients were successfully treated with topical steroids and temporary or permanent ICI withdrawal [122]. A case of lupus nephritis induced by ipilimumab in a patient with melanoma has been reported. The biopsy revealed immunoglobin and complement complexes in the mesangium. The patient had high anti-dsDNA in serum and was managed with ICI discontinuation and prednisolone with nephritis improvement [123]. In conclusion, SLE appears very rarely in the context of cancer immunotherapy; the few reported cases of ICI-induced lupus indicate several differences compared to idiopathic SLE, such as older age and lack of the striking female preponderance.

#### 3.4.2. Systemic Sclerosis (SSc)

Only few cases of scleroderma skin reactions or SSc following ICI treatment have been reported in the literature [26]. The patients reported were on anti-PD1 treatment and presented with skin thickening plus another symptom, such as lung CT imaging abnormalities or Raynaud’s disease. These patients were treated with mycophenolate mofetil or HCQ in combination with prednisolone. Although the underlying mechanism is unknown, it is suggested is that the inflammation triggered by anti-PD1 therapy could lead to local TGFβ activation within the skin triggering a profibrotic cascade [124].

**Figure 1:**
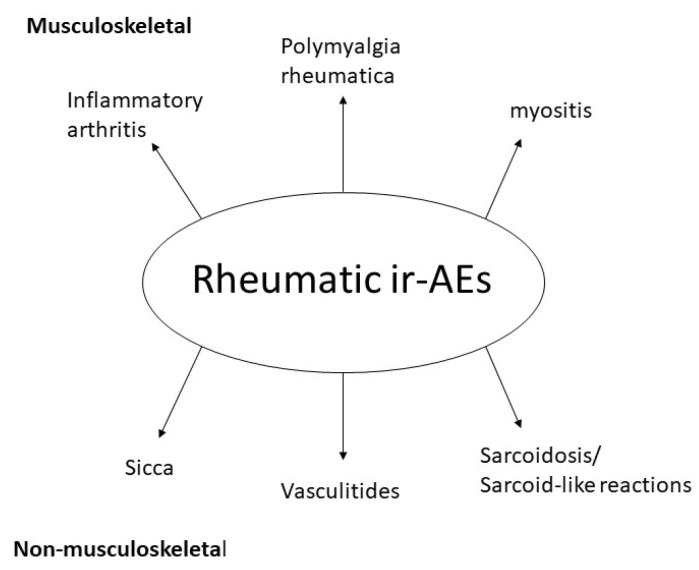
Main immune checkpoint inhibitor (ICI)-induced rheumatic syndromes.

**Figure 2:**
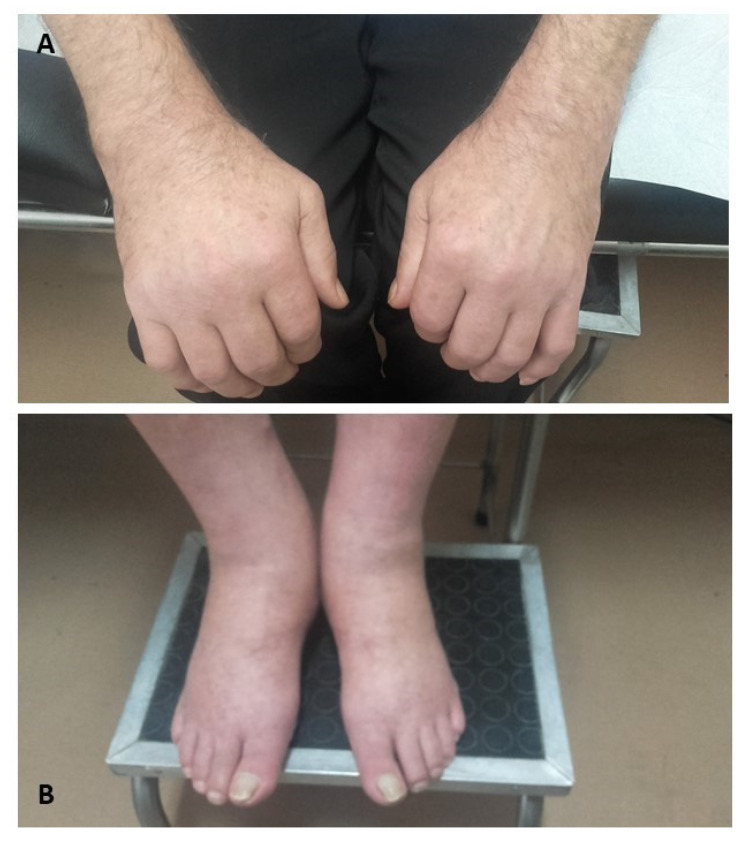
A 65-year-old male with renal cell carcinoma developed diffuse, painful swelling of hands (**A**) and feet (**B**), 3 months following nivolumab treatment (Rheumatology Department of Patras University Hospital photo archive).

An overview of most common ICI-induced rheumatic syndromes is depicted in Figure 1. A patient with ICI-induced musculoskeletal manifestations is shown in Figure 2.

## 4. Discussion

Rheumatic ir-AEs are relatively common and are increasingly recognized. Even though in most cases they are mild/moderate and not life-threatening, they should be diagnosed and managed early since they are associated with pain and functional impairment. Moreover, in light of newer evidence that musculoskeletal ir-AEs may correlate with a favorable tumor response, the appropriate management of these patients by a multidisciplinary team comprised of oncologists and rheumatologists appears to be of critical significance; in this way, patients may be treated effectively and immunotherapy could be maintained.

Rheumatic ir-AEs can be classified as musculoskeletal and non-musculoskeletal. Overall, musculoskeletal manifestations induced by ICI therapy are relatively common, developing in approximately 5%–7.7% of patients according to the prospective studies reported so far. The clinical syndromes most often described are inflammatory arthritis, PMR and myositis. A rheumatology referral is needed in all cases of new-onset inflammatory arthralgia or joint swelling. In most cases of inflammatory arthritis and PMR, ICI therapy may be continued; the majority of patients respond to steroids of less than 20 mg of prednisolone. ICI-induced myositis may have atypical features such as muscle pain or even normal CPK levels. Usually it is the most severe musculoskeletal manifestation induced by ICIs and frequently requires permanent immunotherapy cessation and treatment with high-dose steroids.

The most common forms of non-musculoskeletal rheumatic ir-AEs are sicca, vasculitides and sarcoidosis/sarcoid-like reactions. Cases of SLE and SSc are extremely rare. Of note, ICI-induced rheumatic syndromes appear to have significant differences from their idiopathic counterparts. For example, in most cases, ICI-induced inflammatory arthritis does not have the typical features of RA. Table 1 summarizes all major differences between ICI-induced syndromes and idiopathic counterparts. These data point to the direction that rheumatic ir-AEs are indeed novel clinical entities that should be thoroughly investigated and may not share common pathogenetic mechanisms with idiopathic rheumatic diseases.

In conclusion, the management of ir-AEs in oncologic patients ideally requires a multidisciplinary team with the participation of a rheumatologist. Rheumatic ir-AEs are relatively common; these manifestations should be managed effectively in order to preserve the quality of life of patients with cancer. Large-scale, prospective studies are needed to better delineate the prevalence and clinical characteristics of ICI-induced rheumatic syndromes and to develop relevant therapeutic guidelines.

## Figures and Tables

**Table 1:** Main differences between ICI-induced rheumatic syndromes and their idiopathic counterparts.

**RA**	**ICI-Induced Arthritis**
Usually involves small joints of the hands in a symmetrical fashion	May manifest as mono, oligo or polyarthritis
Synovium is primarily targeted	Apart from synovitis, myo-fasciitis may be prominent
Responds to steroids but treatment with DMARDs is always needed	Good response to steroids DMARD needed when relapse occurs during steroid tapering
**PMR**	**ICI-Induced PMR**
Aching and stiffness in the shoulder and pelvic girdles are typical symptoms	Joint involvement, including knees and hands, may occur
High inflammatory markers are a diagnostic criterion	Absence of increased inflammatory markers is reported in several cases
Responds to low dose of steroids (prednisolone, 20 mg/daily)	Aggressive treatment with higher doses of steroids may be needed
**Polymyositis/Dermatomyositis**	**ICI-Induced Myositis**
Typical clinical presentation involves proximal muscle weakness, without associated muscle pain, sparing facial muscles Dermatomyositis exhibits typical rash	May present with myalgia and oculomotor symptoms, while typical rash is usually absent
Increase in muscle enzymes and autoantibodies against nuclear or cytoplasmic antigens aid diagnosis	May exhibit significant increase in muscle enzymes, albeit normal in a subset of patients Autoantibodies are usually absent
High-dose steroids are the mainstay of treatment Additional immunosuppression is needed in resistant disease and extramuscular features such as ILD	High-dose steroids are usually required, even though milder clinical phenotypes respond well to moderate doses Increased frequency of concurrent myasthenia and/or cardiac involvement is reported and may warrant additional immunosuppression
**Systemic Vasculitides**	**ICI-Induced Vasculitis**
High inflammatory burden is typical Autoantibodies, such as ANCA, can aid diagnosis in subsets of the disease	Inflammatory markers are commonly increased, but autoantibody positivity is rare
**Sjogren Syndrome**	**ICI-Induced Sicca**
Striking female preponderance	Male predominance in some case series
Specific autoantibodies are typically positive	Absence of autoantibodies is reported in most cases
Dry eyes and dry mouth are the most frequent complaints	Dry mouth is the most prominent symptom
**SLE**	**ICI-Induced Lupus**
Typically affects females of childbearing age Antinuclear antibodies are almost always positive	Older age, lack of striking female predominance and absence of autoantibodies are reported

ICI = immune checkpoint inhibitor, RA = rheumatoid arthritis, PMR = polymyalgia rheumatica, ILD = interstitial lung disease, ANCA = antineutrophil cytoplasmic antibodies, DMARDs = disease-modifying antirheumatic drugs, SLE = systemic lupus erythematosus.
